# Predictive model for high‐frequency microsatellite instability in colorectal cancer patients over 50 years of age

**DOI:** 10.1002/cam4.1088

**Published:** 2017-05-23

**Authors:** Kenji Fujiyoshi, Tatsuro Yamaguchi, Miho Kakuta, Akemi Takahashi, Yoshiko Arai, Mina Yamada, Gou Yamamoto, Sachiko Ohde, Misato Takao, Shin‐ichiro Horiguchi, Soichiro Natsume, Shinsuke Kazama, Yusuke Nishizawa, Yoji Nishimura, Yoshito Akagi, Hirohiko Sakamoto, Kiwamu Akagi

**Affiliations:** ^1^ Division of Molecular Diagnosis and Cancer PreventionSaitama Cancer CenterSaitamaJapan; ^2^ Department of SurgeryKurume UniversityFukuokaJapan; ^3^ Department of SurgeryTokyo Metropolitan Cancer and Infectious Diseases Center Komagome HospitalTokyoJapan; ^4^ Hereditary Tumor Research ProjectTokyo Metropolitan Cancer and Infectious Diseases Center Komagome HospitalTokyoJapan; ^5^ Center for Clinical EpidemiologyGraduate School of Public Health Planning OfficeSt. Luke's International UniversityOMURA Susumu & Mieko MemorialSt. Luke's Center for Clinical AcademiaTokyoJapan; ^6^ Department of Digestive Tract and General SurgerySaitama Medical CenterSaitama Medical UniversitySaitamaJapan; ^7^ Department of PathologyTokyo Metropolitan Cancer and Infectious Diseases Center Komagome HospitalTokyoJapan; ^8^ Division of Gastroenterological SurgerySaitama Cancer CenterSaitamaJapan

**Keywords:** Colorectal cancer, immune checkpoint inhibitor, Lynch syndrome, microsatellite instability, mismatch repair deficiency, predictive model, universal tumor screening

## Abstract

Microsatellite instability (MSI) is an important biomarker for screening for Lynch syndrome, and also of response to immune checkpoint inhibitors. The aim of this study is to create a predictive model to determine which elderly patients with colorectal cancer (CRC) should undergo MSI and/or immunohistochemistry testing on the basis of clinicopathological data. We analyzed a test cohort of CRC patients aged ≥50 years (*n* = 2219) by multivariate logistic regression analyses to identify predictors of high‐frequency MSI (MSI‐H). The created prediction model was validated in an external cohort (*n* = 992). The frequency of MSI‐H was 5.5% among CRC patients aged ≥ 50 years. The following five predictors of MSI‐H were identified in the test cohort: female (1 point), mucinous component (2 points), tumor size ≥ 60 mm (2 points), location in proximal colon (3 points), and *BRAF* mutation (6 points). The area under curve (AUC) in the receiver‐operating characteristic (ROC) analysis of this prediction model was 0.832 (95% confidence interval: 0.790–0.874). The sensitivity and specificity were 74.4% and 77.7%, respectively, for a cut‐off score of 4 points. The receiver‐operating characteristic curve of the validation cohort also showed an AUC of 0.856 (95% CI: 0.806–0.905). This prediction model is useful to select elderly CRC patients who should undergo MSI testing, and who may benefit from treatment with 5‐FU‐based adjuvant chemotherapy and cancer immunotherapy.

## Introduction

Colorectal cancer (CRC) is the most common gastrointestinal cancer and one of the leading causes of cancer‐related deaths worldwide 1. At least two types of genomic instability are involved in CRC development, chromosomal instability, and microsatellite instability (MSI) 2. Microsatellite instability is an important biomarker to screen for Lynch syndrome (LS) 3. The consensus criteria for the diagnostic algorithm for LS are based on selecting patients who fulfill the Amsterdam criteria or any of the revised Bethesda guidelines (RBG), followed by MSI testing and/or immunohistochemistry (IHC) staining of MMR proteins 4. RBG is a set of clinical criteria that consist of age at CRC diagnosis, past history of cancers, family history of LS‐related cancers, and histopathological findings in CRC. Although the selection of patients by RBG is inexpensive and does not require technical expertise, relatively low sensitivity 5 and have been shown to missing a substantial number of LS diagnosis. Therefore, universal tumor screening (UTS), which entails routine MSI (and/or IHC) testing for all CRCs, has been recommended in Western countries 6, 7. In fact, several studies have shown that UTS is cost effective 8, 9, 10. Implementing UTS in Asia including Japan is still controversial because the frequency of MSI‐H CRC is low (about 6% of CRCs) 11, 12, 13 and the frequency of LS and the utility of RBG remain unknown.

Meanwhile, recent studies have demonstrated that microsatellite instability is also an important prognostic biomarker for CRC 14 and may be useful as patient selection marker for adjuvant chemotherapy 15, 16 and immune checkpoints inhibitors 17. Advanced CRC with MSI‐H demonstrated a high response rate to treatment with a programmed death‐1 (PD‐1) inhibitor, namely, an immune checkpoint inhibitor 17, and benefit of 5‐FU‐based adjuvant chemotherapy in patient with stage II or III MSI‐H CRC was not observed 18. Thus, MSI status is an important predictor of clinical benefit from these agents. This highlights why not only LS‐related CRC but also sporadic MSI‐H CRC should be identified. The majority of CRCs with MSI‐H are sporadic and develop as a result of silencing of *MLH1* by hypermethylation of its promoter 19, 20. All CRC cases less than 50 years of age are selected for MSI testing by RBG, but there are no criteria for CRC cases over 50 years of age to conduct MSI testing. Considering that most CRC patients are over 50 years of age and the fact that most of them are not MSI‐H, cost‐effective algorithm is required for elderly CRC patients.

To address this problem, we have developed a model to predict which CRC patients older than 50 years should undergo MSI testing on the basis of clinical and pathological data.

## Materials and Methods

### Patients and samples

A total of 2387 consecutive patients with surgically resected CRC at the Saitama Cancer Center were enrolled in the test cohort from July 1999 to September 2014. A total of 1,648 consecutive patients with surgically resected CRC at Tokyo Metropolitan Cancer and Infectious Diseases Center, Komagome Hospital, were enrolled in the validation cohort from January 2008 to August 2016. Patients with a history of preoperative radiotherapy or chemotherapy, inflammatory bowel disease, or a history of familial adenomatous polyposis were excluded. Clinical and pathological information was obtained from medical records.

Tumor tissues were resected surgically and stored at 4°C until time of sampling. A small piece of primary tumor and paired normal colorectal tissue was taken macroscopically by surgeons within 4 h after resection and stored at −80°C immediately. Histopathology was performed by pathologists using remnant tissues of sampling. Hematoxylin and eosin‐stained slides of tumor tissues were reviewed by pathologists to evaluate mucinous component. Tumors were considered mucinous component positive if more than 10% of their volume consisted of mucin, and were considered as mucinous adenocarcinoma if more than 50%.

Informed consent was obtained from all patients included in this study. The study was approved by the Ethics Committees of Saitama Cancer Center (No. 476) and Tokyo Metropolitan Cancer and Infectious Diseases Center, Komagome Hospital (No. 1433, No.1616). All procedures performed in this study were conducted in accordance with the ethical standards of Institutional and National Research Committees and with the 1964 Helsinki Declaration and its later amendments.

### Analysis of *KRAS*/*RAF* mutation

Genomic DNA was extracted from fresh‐frozen tissue samples using the standard phenol–chloroform extraction method. *KRAS* mutations in exons 2, 3, and 4 were analyzed by high‐resolution melting analysis, using a Rotor‐Gene Q (Qiagen, Hilden, Germany) 11, 21, and *BRAF* mutations in exon 15 (codon 600) were detected by either polymerase chain reaction (PCR)‐restriction fragment length polymorphisms or high‐resolution melting analysis, as described previously 22.

### Analysis of microsatellite instability

MSI analysis was performed using fluorescence‐based PCR, as described previously 23. MSI status was determined using five Bethesda markers (BAT25, BAT26, D5S346, D2S123, and D17S250) and classified as MSI‐H (two or more markers demonstrated to be unstable), MSI‐low (MSI‐L; only one marker unstable), and MSS (no markers unstable). MSI‐positive markers were reexamined at least twice to confirm the results. MSI‐L was included with MSS in this study.

### Analysis of *MLH1* promoter hypermethylation

All MSI‐H CRCs in the test cohort were analyzed for *MLH1* promoter methylation status by methylation‐specific PCR or combined bisulfite restriction analysis, as described previously 22.

### Statistical analysis

Patient characteristics were compared using *t*‐tests for continuous variables and *χ* ^2^ or Fisher's exact tests for categorical variables. To select final predictors, all candidate predictors with a *P* < 0.1 in univariate analysis were included in a multivariate logistic regression model. Scores for each predictor were obtained based on the beta value from the final prediction model. Final predictive scores were integers of standardized beta. A receiver‐operating characteristic (ROC) curve was drawn and the area under curve (AUC) was obtained 24. The dataset from Tokyo Metropolitan Cancer and Infectious Diseases Center Komagome Hospital was analyzed for external validation. All analyses were carried out using SPSS software package version 22.0 (SPSS, Inc., IBM Corp., Armonk, NY).

## Results

### Patient characteristics

Data were not available for 7 patients in the test cohort and 54 in the validation cohort. Data for 2380 patients in the test cohort and 1094 in the validation cohort were therefore included in the following analysis.

There were no significant differences in gender, *BRAF* mutation, and MSI status between the two cohorts. Mean age at diagnosis of CRC and location were significantly different, but were not clinically significant. Advanced stage, larger tumor size, mucinous component, and wild‐type *KRAS* were significantly more frequent in the validation cohort (Table 1).

**Table 1:** Baseline characteristics of test and validation cohorts

	Test	Validation	*P*
	*n* = 2380 (%)	*n* = 1094 (%)	
Gender			
Female	988 (41.5)	473 (43.2)	0.339
Male	1392 (58.5)	621 (58.2)	
Age at diagnosis of CRC			
Mean ± SD	65.0 ± 10.2	66.3 ± 11.6	0.02
Location			
Proximal	704 (29.6)	286 (26.1)	0.037
Distal	1676 (70.4)	808 (73.9)	
Tumor size			
Mean ± SD(mm)	45.6 ± 23.9	52.1 ± 23.9	<0.001
TNM stage			
0–I	532 (22.4)	124 (11.4)	<0.001
II	745 (31.3)	374 (34.2)	
III	712 (29.9)	367 (33.5)	
IV	391 (16.4)	229 (20.9)	
Mucinous component			
−	2086 (87.6)	902 (82.4)	<0.001
+	294 (12.4)	192 (17.6)	
*KRAS*			
Wild	1370 (57.6)	784 (71.7)	<0.001
Mutant	1010 (42.4)	310 (28.3)	
*BRAF*			
Wild	2272 (95.5)	1042 (95.2)	0.778
Mutant	108 (4.5)	52 (4.8)	
MSI status			
MSI‐H	139 (5.8)	60 (5.5)	0.675
MSS	2241 (94.2)	1034 (94.5)	

Proximal, cecum to transverse colon; Distal, splenic flexure to rectum; TNM, tumor node metastasis; MSI‐H, high‐frequency microsatellite instability; MSS, microsatellite stable.

### Univariate analysis in the test cohort

There were 2219 patients aged ≥ 50 years in the test cohort and 992 in the validation cohort. The results of univariate analysis in the test cohort are shown in Table 2. CRCs with MSI‐H were more frequently associated with female sex (vs. MSS, *P *<* *0.001), location in the proximal colon (vs. MSS, *P *<* *0.001), large tumor size (vs. MSS, *P *<* *0.001), mucinous component (vs. MSS, *P *<* *0.001), and *BRAF* mutation (vs. MSS, *P *<* *0.001), and were less frequently associated with *KRAS* mutation (vs. MSS, *P *=* *0.01). The mean age at CRC diagnosis was similar in patients with MSI‐H and MSS (*P *=* *0.07). Tumor size of MSI‐H CRCs was significantly larger than that of MSS CRCs in proximal (58.9 mm vs. 45.1 mm, *P *=* *0.001) and distal (56.8 mm vs. 44.4 mm, *P *=* *0.045). Thus, MSI‐H CRCs are larger than MSS CRCs regardless of tumor location.

**Table 2:** Characteristics of CRC patients aged ≥50 years in relation to MSI

	MSI‐H	MSS	*P*
	*n* = 121 (%)	*n* = 2098 (%)	
Gender			
Female	70 (7.7)	841 (92.3)	<0.001
Male	51 (3.9)	1257 (96.1)	
Age at diagnosis of CRC			
Mean ± SD	68.1 ± 9.56	66.4 ± 8.44	0.07
Location			
Proximal	86 (12.9)	582 (87.1)	<0.001
Distal	35 (2.3)	1516 (97.7)	
Tumor size			
Mean ± SD(mm)	58.3 ± 35.9	44.6 ± 22.5	<0.001
TNM stage			
0‐I	28 (5.6)	471 (94.4)	<0.001
62 (8.8)	642 (91.2)		
21 (3.2)	635 (96.8)		
10 (2.8)	350 (97.2)		
Mucinous component			
−	79 (4.1)	1868 (95.9)	<0.001
+	42 (15.4)	230 (84.6)	
*KRAS*			
Wild	82 (6.5)	1183 (93.5)	0.01
Mutant	39 (4.1)	915 (95.9)	
*BRAF*			
Wild	75 (3.5)	2046 (96.5)	<0.001
Mutant	46 (46.9)	52 (53.1)	

Proximal, cecum to transverse colon; Distal, splenic flexure to rectum; TNM, tumor node metastasis; MSI‐H, high‐frequency microsatellite instability; MSS, microsatellite stable.

### Multivariate analysis for predictive model

According to multivariate analysis of tumor size, CRC with MSI‐H was more frequent in patients with tumors 0–19 mm (hazard ratio [OR] = 2.79, *P *=* *0.01, 95% confidence interval [CI]: 1.26–6.19), 20–29 mm (OR = 2.40, *P = *0.02, 95% CI: 1.15–5.02), and ≥ 60 mm (OR = 4.83, *P *<* *0.001, 95% CI: 2.52–9.27), compared with the reference size (30–39 mm) (Table 3).

**Table 3:** Multivariate analysis according to tumor size

	Beta	*P*	OR	95% CI	
				Lower	Upper
0–19 (mm)	1.03	0.01	2.79	1.26	6.19
20–29 (mm)	0.88	0.02	2.40	1.15	5.02
30–39 (mm)	—	—	Ref	—	—
40–49 (mm)	0.35	0.38	1.42	0.64	3.13
50–59 (mm)	0.28	0.52	1.32	0.57	3.09
60‐ (mm)	1.58	0.00	4.83	2.52	9.27

**Figure 1:**
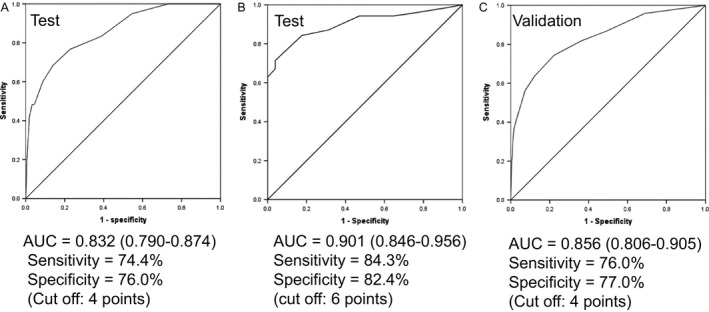
ROC curves of test and validation cohorts. (A) ROC curve of test cohort. (B) ROC curves of *MLH1* promoter methylation in the test cohort of CRC with MSI‐H. (C) ROC curve of validation cohort. ROC curves of test and validation cohorts were identical.

**Figure 2:**
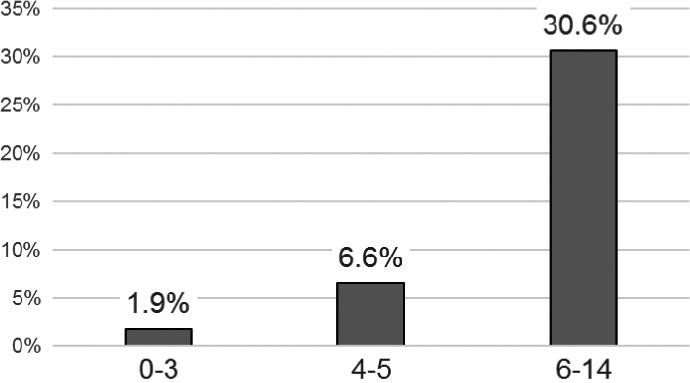
Frequency of CRC with MSI‐H. Frequency of CRC with MSI‐H according to prediction score in the test cohort. The frequency of CRC with MSI‐H increased with increasing score. ROC, receiver‐operating characteristic.

Multivariate logistic regression analysis was conducted including all of the above candidate predictors (gender, location, tumor size 0–19 mm, tumor size 20–29 mm, tumor size ≥ 60 mm, mucinous component, *KRAS* mutation, and *BRAF* mutation). *BRAF* mutation, female sex, mucinous component, location in proximal colon, and size ≥ 60 mm were subsequently selected as predictors based on a *P* < 0.05. The final model of MSI predictors is shown in Table 4. Female sex scored 1 point, mucinous component and size ≥ 60 mm scored 2 points each, proximal location scored 3 points, and *BRAF* mutation scored 6 points. The sum of the scores for each patient was calculated and a ROC curve of the test cohort was constructed. The AUC of the prediction model was 0.832 (95% CI: 0.790–0.874). The sensitivity and specificity were 74.4% and 77.7%, respectively, for a cut‐off value of 4 points (Fig. 1A). The frequency of CRC with MSI‐H for each score is shown in Table 5. The frequencies of MSI‐H were 1.9% (0–3 points), 6.6% (4–5 points), and 30.6% (6–14 points) (Fig. 2). MSI‐H CRC with *MLH1* promoter hypermethylation was more frequent (59/70, 84.3%) in patients with a score ≥6, whereas MSI‐H CRC with unmethylated *MLH1* was more frequent (42/51, 82.4%) in those with a score ≤ 5 (*P *<* *0.001) (Table 6). The AUC for *MLH1* promoter‐methylated CRC with MSI‐H was 0.901 (0.846–0.956) (Fig. 1B). Sixteen LS patients were included in this test cohort, and 93.3% (14/15) scored ≤ 5.

**Table 4:** Multivariate analysis of factors predicting CRC with MSI‐H in patients aged ≥50 years

	Beta	Odds	95% CI		Score
Female	0.44	1.56	1.02	2.38	1
Size ≥ 60 mm	1.01	2.75	1.80	4.20	2
Mucinous	0.76	2.13	1.32	3.42	2
Proximal	1.32	3.76	2.41	5.86	3
*BRAF* mutant	2.59	13.33	8.01	22.20	6

**Table 5:** Frequency of CRC with MSI‐H among patients according to prediction score

Score	MSI‐H/Total (%)	MSI‐H (*N* = 121)	Total (*N* = 2219)	MSI‐H (*N* = 121)		LS (*N* = 16)
				*MLH1*‐M (*N* = 70)	un‐M (*N* = 51)	
0	0.8	5	657	1	4	1
1	2.6	11	424	2	9	4
2	2.2	6	276	1	5	1
3	3.0	9	305	0	9	1
4	5.8	13	224	5	8	4
5	8.1	9	111	2	7	4
6	16.2	16	99	9	7	0
7	16.7	3	18	3	0	0
8	18.5	5	27	3	2	1
9	38.9	7	18	7	0	0
10	55.6	10	18	10	0	0
11	40.0	4	10	4	0	0
12	73.7	14	19	14	0	0
13	60.0%	3	5	3	0	0
14	75.0%	6	8	6	0	0

*MLH1*‐M, *MLH1* promoter hypermethylated; un‐M, *MLH1* promoter unmethylated; LS, Lynch syndrome.

**Table 6:** Presence of *MLH1* promoter hypermethylation in CRC with MSI‐H

	*MLH1*‐M	un‐M	*P*
	(*N* = 70)	(*N* = 51)	
0–5 points	11	42	<0.001
6–14 points	59	9	

Sensitivity = 84.3%, specificity = 82.4%.

*MLH1*‐M, *MLH1* promoter hypermethylated; un‐M, *MLH1* promoter unmethylated.

### Validation

We evaluated the accuracy of this model in an external validation cohort from Tokyo Metropolitan Cancer and Infectious Diseases Center Komagome Hospital. The ROC curve of the validation cohort had an AUC of 0.856 (95% CI: 0.806–0.905). The sensitivity and specificity were 76.0% and 77.0%, respectively, for a cut‐off score of 4 points (Fig. 1C).

## Discussion

In this study, we developed a model to predict MSI‐H CRC patients aged ≥ 50 years based on data from two CRC cohorts. The model demonstrated relatively high sensitivity and specificity, especially for sporadic CRC with MSI‐H, and was robust according to external validation.

According to this model, 30.6% of CRC patients with a prediction score ≥ 6 had MSI‐H, whereas 98.1% (1631/1662) with a score ≤ 3 had MSS. This model could thus reduce the time and cost involved in identifying MSI‐H CRC. The results suggest that MSI testing should be strongly recommended in patients with a score ≥ 6, should ideally be carried out in those with a score of 4 or 5, and if possible, in those with a score ≤ 3. Furthermore, *MLH1* promoter‐hypermethylated CRCs were more frequent among patients with a score ≥ 6, whereas unmethylated ones, including LS‐related CRCs, were more frequent in those with a score ≤ 5. This model could therefore also anticipate the presence of *MLH1* promoter hypermethylation after MSI testing.

MSI status is an important biomarker for prognosis, and potential patient selection biomarker for adjuvant chemotherapy and immune checkpoints inhibitor. Frequency of MSI‐H is very low in stage III and IV CRCs, 3.2% and 2.8%, respectively (Table 2). Therefore, appropriate selection from stage III and IV CRCs is required for cost effectiveness. Among stage III and IV CRCs (*N* = 1016), frequency of MSI‐H that scored 0–3 points is 0.95% (7/740), scored 4–5 points is 3.2% (5/155), and scored 6 points or more is 15.7% (19/121), and is enriched about fivefold in ≥6 points group. That is, 61.3% (19/31) of CRCs with MSI‐H is included in ≥6 points group. Thus, this enrichment will help decision making of MSI testing.

Several predictive models have previously been reported 25, 26, 27, including pathological findings such as Crohn‐like reaction, tumor‐infiltrating lymphocytes, cribriform, Ki67 index, and p53 overexpression. These predictive models may be highly sensitive and specific, but the requirement for detailed pathological diagnosis puts a big burden on the pathologists.

The current predictive model identified five predictors by multivariate logistic regression analysis: *BRAF* mutation, female sex, location in the proximal colon, and tumor size ≥ 60 mm; will be available as a result of routine medical treatment. Information on mucinous component will also be available if a pathologist helps to evaluate this. This is the first predictive model to include *BRAF* mutation as a predictive factor. *BRAF* mutation analysis is currently not so common in CRC patients, but multigene testing including *KRAS*,* NRAS*, and *BRAF* by luminex‐based multiplex assay will be available soon in Japan, and the European Society for Medical Oncology consensus guidelines recommend *BRAF* testing as grade B 28. In addition, multigene testing by Next‐Generation Sequencing will become increasingly utilized in many countries to select appropriate cancer therapy 29. *BRAF* mutation is significantly associated with MSI‐H, especially MSI‐H with *MLH1* promoter hypermethylation 20, 30, 31, 32. *BRAF* mutation was the strongest predictor in our model, suggesting that this model could select more CRCs with hypermethylated, compared with CRCs with unmethylated *MLH1* promoters. The incidence of MSI‐H CRCs is known to be increased in older women, in tumors located in the proximal colon, and among mucinous component tumors 33. CRC with *MLH1* promoter hypermethylation is also more common in women 19. The frequency of *BRAF* mutations varies widely from 1.1% to 15.3% worldwide 29, 34, 35, 36, 37, 38. The *BRAF* V600E mutation frequency of 4.5–4.8% observed in this study is consistent with various Asian studies (1.1% to 4.9%), but is slightly lower than several Western studies (7.0–15.3%). In contrast, it has recently been reported that *BRAF* non‐V600E mutations were found more frequently in Asian than in Western 39, 40, 41. However, it remains unknown whether *BRAF* non‐V600E mutations correlate with MSI‐H CRC or not.

Similar to our predictive model, Hyde et al. also included proximal location and mucinous component 26, and Colomer et al. included mucinous component and tumor size in their models 27. Considering tumor size, CRCs with MSI‐H are known to be larger than CRCs with MSS 14, 31. Our data also showed significant difference between MSI‐H and MSS, regardless of tumor location. We used a cut‐off value for tumor size of ≥60 mm, compared with > 65 mm in Colomer et al. model 27 and > 50 mm in Batur et al. report 42. It is difficult to judge the optimal cut‐off for tumor size as a predictor because tumor size changes according to the stage or timing of operation. However, typical CRCs do not grow to ≥60 mm 43, 44, and the cut‐off values were therefore appropriate based on the characteristic large size of CRCs with MSI‐H. It is interesting to note that the frequency of MSI‐H CRC is higher in the group of tumors that are smaller in size (Table 3). One of the reasons for this may be that the high‐risk group, such as LS or suspected LS cases, had taken regular colonoscopic surveillance. This may lead to diagnosis of CRC at an earlier stage. Further investigations are required to clarify this observation.

Regarding LS, 25 patients with LS‐related CRCs were enrolled across all ages in the test cohort, including 16 (64%) among patients ≥ 50 years, of whom 43% (7/16) scored ≤ 3.

CRC patients with MSI‐H could not be perfectly isolated in the test cohort. Thirty‐one CRCs with MSI‐H were included among 1662 cases that scored ≤ 3, accounting for 25.6% (31/121) of all CRCs with MSI‐H. Among 1886 CRCs with *BRAF* wild‐type and no mucinous component, 51 (2.7%) CRC were MSI‐H, 31 cases scored 0–3 points, 10 cases scored 4–5 points, and 10 cases scored ≥ 6 points. The current predictive model could identify 39.2% (20/51) of MSI‐H CRC with *BRAF* wild‐type and no mucinous component. Inamura et al. reported that the existence of signet‐ring cell component is associated with MSI‐H 45. If our prediction model incorporates signet‐ring cell component, prediction rate may be improved.

There were some limitations to this study. Tissue samples were not assessed for quality microscopically to evaluate presence of cancer cells.

In conclusion, we developed a predictive model to determine the need for MSI testing among CRC patients aged ≥ 50 years. This model can help to identify those CRCs with MSI‐H, especially sporadic CRC with MSI‐H.

We expect that this predictive model will be useful in clinical situations, such as determining which patients should be recommended for indication for 5‐fluorouracil‐based adjuvant therapy and to identify those patients who may derive therapeutic benefit from immune checkpoint inhibitors.

## Conflict of Interest

The authors have no disclosures to make.
